# Modelling and Analyzing Virus Mutation Dynamics of Chikungunya Outbreaks

**DOI:** 10.1038/s41598-019-38792-4

**Published:** 2019-02-27

**Authors:** Xiaomei Feng, Xi Huo, Biao Tang, Sanyi Tang, Kai Wang, Jianhong Wu

**Affiliations:** 1 School of mathematics and information sciences, Shaanxi Normal University, Xi’an, 710062 People’s Republic of China; 2 School of mathematics and information technology, Yuncheng University, Yuncheng, 044000 People’s Republic of China; 3 Department of Mathematics, University of Miami, Coral Gables, FL 33124-4250 USA; 4 Laboratory for Industrial and Applied Mathematics, Faculty of Sciences, York University, Toronto, ON M3J1P3 Canada; 5 Department of Medical Engineering and Technology, Xinjiang Medical University, Urumqi, 830011 People’s Republic of China

## Abstract

Chikungunya fever, caused by chikungunya virus (CHIKV) and transmitted to humans by infected *Aedes* mosquitoes, has posed a global threat in several countries in 2015. Recent outbreaks in La Réunion, Italy and China are related with a new variant of CHIKV with shorter extrinsic incubation period in contaminated mosquitoes, but the role of this new variant on the spread of chikungunya fever is unclear. We develop a mathematical model that incorporates the virus mutation dynamics in the transmission of CHIKV among mosquitoes and humans. Our numerical simulations show that a substantial virus mutation rate combined with high virus transmission probabilities from mosquito to human, could result in sustainable chikungunya fever outbreaks. Further, we apply Markov Chain Monte Carlo sampling method to fit our model to the 2007 chikungunya fever outbreak data in North-Eastern Italy where the mutant strain was detected. We conclude that the basic reproduction number might be underestimated without considering the mutation dynamics, and our estimation shows that the basic reproduction number of the 2007 Italy outbreak was ${\pmb{\mathscr{R}}}_{{\bf{0}}}$ = 2.035[95%Cl: 1.9424 - 2.1366]. Sensitivity analysis shows that the transmission rate of the mutant strain from mosquitoes to human is more influential on ${\pmb{\mathscr{R}}}_{{\bf{0}}}$ than the shortened extrinsic incubation period. We conclude that the virus mutation dynamics could play an important role in the transmission of CHIKV, and there is a crucial need to better understand the mutation mechanism.

## Introduction

Chikungunya fever is a vector-borne disease that is transmitted to humans by *Aedes* mosquitoes (mainly *Aedes aegypti* and *Aedes albopictus*), and caused by chikungunya virus (CHIKV) which belongs to the *Alphavirus* genus of the family *Togaviridae*^1,2^. The name “chikungunya” comes from a Swahili or Makonde word meaning “to become contorted”, and describes the bent posture of sufferers with joint pain (arthralgia)^3,4^. This disease is characterized by an abrupt onset of fever frequently accompanied by intense asthenia, arthralgia, myalgia, headache, and rash. Because these clinic signs are similar to those of dengue and Zika, misdiagnoses occur frequently. Although most symptoms would resolve, joint pains may persist for several months, or even years, and result in chronic pain and disability. Among all infected people, a large portion of them are symptomatic, and only less than 15% of them have no symptoms. There are currently no licensed vaccines or specific treatments for CHIKV infections.

Chikungunya fever was reported as early as in the 1770s, and CHIKV was first isolated from the serum of a febrile patient during a dengue epidemic which occurred in the Newala district, Tanzania, in 1953^5^. The earliest confirmation of an outbreak in Asia was from the Philippines in 1954. Until the mid-1980s, this endemic strain, called the Asian lineage, had caused outbreaks and sporadic cases in India and Southeast Asia. Three distinct lineages have been identified so far: the west African lineage, the east, central, and southern African lineage (ECSA), and the Asian lineage^6^. After several decades of absence, CHIKV caused a major epidemic in Kenya resulted in 13,500 cases in 2004. In the following two years, the virus rapidly spread to Comoros (2005), La Réunion (one third of the Réunion population were infected during 2005–2006), several other Indian Ocean islands, India (infected more than 1.39 million people in 2006), and parts of Southeast Asia. In 2007, the disease was reported for the first time in Europe, causing a local outbreak in North-Eastern Italy. Until now, CHIKV has been detected in more than 60 countries in Asia, Africa, Europe, and the Americas. A map that depicts the origin, spread, and distribution of CHIKV and the disease vectors can be found in^3^.

CHIKV is a single-stranded, positive-sense RNA virus, enveloped with a spherical shape and icosahedral symmetry. Mutations in the surface envelope glycoproteins E1 at position 226 (E1-A226V) have been identified in recent epidemic strains^3,7^, and the new variant of the virus is identified as a new sublineage known as ECSA-V. The first report of mutation was in the Karnataka state of India^8,9^, it caused an unprecedented magnitude outbreak in the Indian Ocean islands and India from 2004–2007. The same sublineage also caused the outbreak in North-Eastern Italy in 2007 and two outbreaks in Guangdong, China in 2010^10^. Studies have shown that this mutant strain obtains better fitness than the non-mutant strain in *Aedes albopictus* - the abundant mosquito species in Italy, but such species-specific adaptation of this mutant strain was not found in human cells^11^. It was found in the 2007 Italy outbreak that *Aedes albopictus* with ECSA-V are possible to develop a shortened extrinsic incubation period and higher transmission potentials^11–13^, thus it is believed that the appearance of ECSA-V might be contributing to the size of the recent outbreaks in Italy and China^11^. Moreover, it is also worthwhile to notice that ECSA-V with all its special features cannot explain the size of the recent outbreaks in the Americas since this mutation has not been found in the samples collected during these outbreaks.

Various mathematical models have been proposed to describe chikungunya fever transmission, to name a few^14–29^. To the best of our knowledge, none of the previous studies has considered the strain mutation dynamics and estimated its impact through outbreak data. Inspired by these findings, we use mathematical models and data fitting techniques to study the influence of mutation dynamics on the transmission of CHIKV.

The paper is organized as follows: the mathematical model for chikungunya fever is introduced in section 2; the basic reproduction number of the model is calculated in section 3; in section 4, we study the dynamical behaviors of the model as the mutation rate changes; in section 5, we fit the model with the outbreak data in Italy from June 23 to September 14 in 2007. Under the assumption of a linear mutation rate among mosquitoes, sustainable chikungunya outbreaks can be observed with credible parameter values. Our data fitting results show that the basic reproduction number in the 2007 Italy outbreak might be underestimated without considering the mutation dynamics.

## Mathematical model

We stratify the infected mosquitoes and human populations in terms of non-mutant and mutant strains to describe the mutation dynamics. For mosquitoes, the total mosquito population at time $$t$$ is denoted by $${N}_{M}(t)$$, which includes the following epidemiological compartments: susceptible $$({S}_{M})$$, exposed $$({E}_{M1}$$ and $${E}_{M2})$$, infectious $$({I}_{M1}$$ and $${I}_{M2})$$, with sub-index 1 for non-mutant and 2 for mutant strain. Susceptible mosquitoes move to the latent compartment $${E}_{Mi}(i=1,2)$$, upon successful exposure to the respective strain. The mosquito recruitment and death rates are denoted by $${\lambda }_{M}$$ and $${\mu }_{M}$$. Exposed mosquitoes become infectious after the latent period $$\mathrm{1/}{\gamma }_{M1}$$ or $$\mathrm{1/}{\gamma }_{M2}$$. We assume that the virus could mutate in contaminated mosquitoes all through their life time at a rate of *δ*. For humans, we take into account of the asymptomatic and symptomatic infections. The total human population $${N}_{H}$$ includes the following epidemiological compartments: susceptible $$({S}_{H}),$$ exposed $$({E}_{H1}$$ and $${E}_{H2}),$$ asymptomatically infected $$({I}_{H1}^{N},{I}_{H2}^{N})$$, symptomatically infected $$({I}_{H1}$$, $${I}_{H2})$$, and recovered $$({R}_{H1}$$, $${R}_{H2})$$.

**Figure 1:**
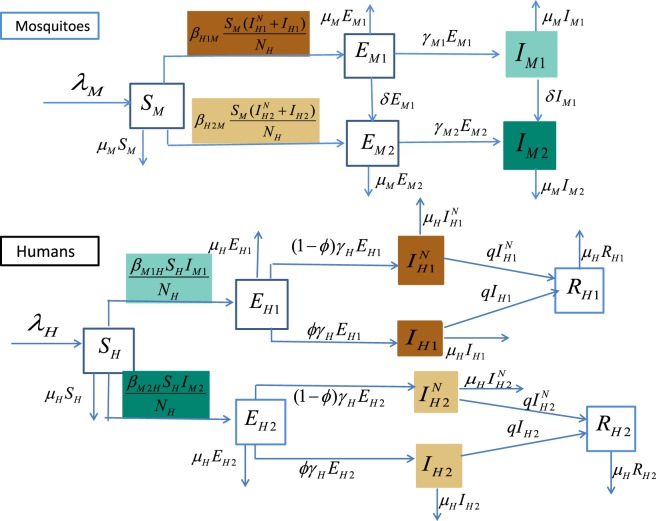
CHIKV transmission flow chart. Subindices 1 and 2 correspond to the non-mutant and mutant strains, human populations are stratified into exposed, asymptomatically/symptomatically infected and recovered compartments. The mutation dynamics is characterized by the flow from the mosquitoes infected with the non-mutant strain to the mosquitoes with the mutant strain. Solid arrows represent the movements of population among compartments. Compartments responsible for the transmission of CHIKV are colored correspondingly to the transmission rates.

We assume that a proportion *ϕ* of exposed individuals will become symptomatic after the latent period $\mathrm{1/}{\gamma }_{H}$, and both symptomatic and asymptomatic individuals have the same probability of transmitting the virus to mosquitoes via each bite. We assume that the virus only mutates in the mosquito population because of the selective pressure that has been demonstrated in mosquito bodies but not been found in humans^11^. It has been shown in^30^ that people experienced the non-mutant CHIKV outbreak in 1975, Cambodia, obtain a lower risk of infection during the outbreak of the mutant strain in 2012. Thus we make a simplified assumption on the existence of cross-immunity between the two strains, *i*.*e*., people recovered from one strain are immune to both of the strains for life time. Following the transmission diagram shown in Fig. 1, our model takes the form in (1).

1 $$\begin{array}{ccc}\displaystyle \frac{d{S}_{M}}{dt} & = & {\lambda }_{M}-b{\beta }_{H1M}\displaystyle \frac{{S}_{M}({I}_{H1}^{N}+{I}_{H1})}{{N}_{H}}-b{\beta }_{H2M}\displaystyle \frac{{S}_{M}({I}_{H2}^{N}+{I}_{H2})}{{N}_{H}}-{\mu }_{M}{S}_{M},\\ \displaystyle \frac{d{E}_{M1}}{dt} & = & b{\beta }_{H1M}\displaystyle \frac{{S}_{M}({I}_{H1}^{N}+{I}_{H1})}{{N}_{H}}-{\gamma }_{M1}{E}_{M1}-{\mu }_{M}{E}_{M1}-\delta {E}_{M1},\\ \displaystyle \displaystyle \frac{d{I}_{M1}}{dt} & = & {\gamma }_{M1}{E}_{M1}-{\mu }_{M}{I}_{M1}-\delta {I}_{M1},\\ \displaystyle \frac{d{E}_{M2}}{dt} & = & b{\beta }_{H2M}\displaystyle \frac{{S}_{M}({I}_{H2}^{N}+{I}_{H2})}{{N}_{H}}-{\gamma }_{M2}{E}_{M2}-{\mu }_{M}{E}_{M2}+\delta {E}_{M1},\\ \displaystyle \frac{d{I}_{M2}}{dt} & = & {\gamma }_{M2}{E}_{M2}-{\mu }_{M}{I}_{M2}+\delta {I}_{M1},\\ \displaystyle \frac{d{S}_{H}}{dt} & = & {\lambda }_{H}-\displaystyle \frac{b{\beta }_{M1H}{S}_{H}{I}_{M1}}{{N}_{H}}-\frac{b{\beta }_{M2H}{S}_{H}{I}_{M2}}{{N}_{H}}-{\mu }_{H}{S}_{H},\\ \displaystyle \frac{d{E}_{H1}}{dt} & = & \displaystyle \frac{b{\beta }_{M1H}{S}_{H}{I}_{M1}}{{N}_{H}}-({\gamma }_{H}+{\mu }_{H}){E}_{H1},\\ \displaystyle \frac{d{I}_{H1}^{N}}{dt} & = & (1-\varphi ){\gamma }_{H}{E}_{H1}-(q+{\mu }_{H}){I}_{H1}^{N},\\ \displaystyle \frac{d{I}_{H1}}{dt} & = & \varphi {\gamma }_{H}{E}_{H1}-(q+{\mu }_{H}){I}_{H1},\\ \displaystyle \frac{d{R}_{H1}}{dt} & = & q({I}_{H2}^{N}+{I}_{H2})-{\mu }_{H}{R}_{H1},\\ \displaystyle \frac{d{E}_{H2}}{dt} & = & \displaystyle \frac{b{\beta }_{M2H}{S}_{H}{I}_{M2}}{{N}_{H}}-({\gamma }_{H}+{\mu }_{H}){E}_{H2},\\ \displaystyle \frac{d{I}_{H2}^{N}}{dt} & = & (1-\varphi ){\gamma }_{H}{E}_{H2}-(q+{\mu }_{H}){I}_{H2}^{N},\\ \displaystyle \frac{d{I}_{H2}}{dt} & = & \varphi {\gamma }_{H}{E}_{H2}-(q+{\mu }_{H}){I}_{H2},\\ \frac{d{R}_{H2}}{dt} & = & q({I}_{H2}^{N}+{I}_{H2})-{\mu }_{H}{R}_{H2}.\end{array}$$

here, $b$ is the average biting rate of the mosquitoes. ${\beta }_{H1M}$ (${\beta }_{H2M}$) is the transmission probability from human with non-mutant strain to mosquitoes (from human with mutant strain to mosquitoes), correspondingly, ${\beta }_{M1H}$ (${\beta }_{M2H}$) is the transmission probability from mosquitoes with non-mutant strain to human (from mosquitoes with mutant strain to human). Symptomatic and asymptomatic individuals have the same recovery rate $q$. The parameter $\delta$ is the virus mutation rate (which is essentially the mutant strain adaptation rate) within the bodies of contaminated mosquitoes. CHIKV usually does not cause deaths of human, therefore, we do not consider the disease related deaths in model (1). ${\lambda }_{H}$ is the human recruitment rate and $\mathrm{1/}{\mu }_{H}$ is the average lifespan of humans. We consider model (1) with non-negative initial conditions and parameter values, and we adopt the ranges of parameters as illustrated in Table 1.

**Table 1:** Model Parameters.

Parameter	Interpretation	Value	Range	Reference
*λ*_*M*_	Recruitment rate of mosquitos (day^−1^)	Estimated	400–5000	^45^
$\mathrm{1/}{\mu }_{M}$	Average lifespan of mosquitoes (days)	28	14–42	^17, 18, 46, 47^
b	Average biting rate of mosquitoes (day^−1^)	0.5	[0.3, 1]	^27, 48, 49^
${\beta }_{H1M}$	Probability of transmission from humans with nonmutant strain to mosquitoes	Estimated	[0.01, 1]	^26, 34^
${\beta }_{H2M}$	Probability of transmission from humans with mutant strain to mosquitoes	Estimated	[0.01, 1]	^26, 34^
$\delta$	Mutation rate of virus inside mosquitoes (day^−1^)	Estimated	[0, 1]	—
$\mathrm{1/}{\gamma }_{M1}$	Extrinsic incubation period in mosquitoes with nonmutant strain (days)	4	2–6	^13, 26^
$\mathrm{1/}{\gamma }_{M2}$	Extrinsic incubation period in mosquitoes with mutant strain (days)	2	—	^12, 13^
${\beta }_{M1H}$	Probability of transmission of nonmutant strain from mosquito to human	Estimated	[0.01, 0.94]	^22, 26^
${\beta }_{M2H}$	Probability of transmission of mutant strain from mosquito to human	Estimated	[0.02, 0.94]	^22, 26^
$\mathrm{1/}{\gamma }_{H}$	Intrinsic incubation period (days)	3	2–4	^17, 44, 46, 47^
$\varphi$	Proportion of symptomatic individuals	0.85	[0.72, 0.97]	^20, 50– 56^
$\mathrm{1/}q$	Infectious period in humans (days)	6	3–7	^18, 26, 47^

It is obvious that system (1) always has a disease free equilibrium ${E}_{0}=(\frac{{\lambda }_{M}}{{\mu }_{M}},0,0,0,0,\frac{{\lambda }_{H}}{{\mu }_{H}},0,0,0,0,0,0,0,0)$. We follow the calculation of basic reproduction number developed in^31^, and rewrite system (1) as the following form

2 $$\dot{x}={\mathscr{F}}(x)-{\mathscr{V}}(x)$$

where $$x={({E}_{M1},{I}_{M1},{E}_{M2},{I}_{M2},{E}_{H1},{I}_{H1}^{N},{I}_{H1},{E}_{H2},{I}_{H2}^{N},{I}_{H2})}^{T}$$ and

$$\begin{array}{cc}{\mathscr{F}}(x)=(\begin{array}{c}b{\beta }_{H1M}\displaystyle \frac{{S}_{M}({I}_{H1}^{N}+{I}_{H1})}{{N}_{H}}\\ 0\\ b{\beta }_{H2M}\displaystyle \frac{{S}_{M}({I}_{H2}^{N}+{I}_{H2})}{{N}_{H}}\\ 0\\ \displaystyle \frac{b{\beta }_{M1H}{S}_{H}{I}_{M1}}{{N}_{H}}\\ 0\\ 0\\ \displaystyle \frac{b{\beta }_{M2H}{S}_{H}{I}_{M2}}{{N}_{H}}\\ 0\\ 0\end{array}), & {\mathscr{V}}(x)=(\begin{array}{c}({\gamma }_{M1}+{\mu }_{M}+\delta ){E}_{M1}\\ -{\gamma }_{M1}{E}_{M1}+({\mu }_{M}+\delta ){I}_{M1}\\ -\delta {E}_{M1}+({\gamma }_{M2}+{\mu }_{M}){E}_{M2}\\ -{\gamma }_{M2}{E}_{M2}+{\mu }_{M}{I}_{M2}-\delta {I}_{M1}\\ ({\gamma }_{H}+{\mu }_{H}){E}_{H1}\\ -(1-\varphi ){\gamma }_{H}{E}_{H1}+(q+{\mu }_{H}){I}_{H1}^{N}\\ -\varphi {\gamma }_{H}{E}_{H1}+(q+{\mu }_{H}){I}_{H1}\\ ({\gamma }_{H}+{\mu }_{H}){E}_{H2}\\ -(1-\varphi ){\gamma }_{H}{E}_{H2}+(q+{\mu }_{H}){I}_{H2}^{N}\\ -\varphi {\gamma }_{H}{E}_{H2}+(q+{\mu }_{H}){I}_{H2}\end{array}).\end{array}$$

Calculate the derivatives of ${\mathcal F}$ and ${\mathscr{V}}$ at the disease-free equilibrium *E*_0_ we have

$$F=(\begin{array}{cccccccccc}0 & 0 & 0 & 0 & 0 & b{\beta }_{H1M}\frac{{S}_{M}}{{N}_{H}} & b{\beta }_{H1M}\frac{{S}_{M}}{{N}_{H}} & 0 & 0 & 0\\ 0 & 0 & 0 & 0 & 0 & 0 & 0 & 0 & 0 & 0\\ 0 & 0 & 0 & 0 & 0 & 0 & 0 & 0 & b{\beta }_{H2M}\frac{{S}_{M}}{{N}_{H}} & b{\beta }_{H2M}\frac{{S}_{M}}{{N}_{H}}\\ 0 & 0 & 0 & 0 & 0 & 0 & 0 & 0 & 0 & 0\\ 0 & b{\beta }_{M1H} & 0 & 0 & 0 & 0 & 0 & 0 & 0 & 0\\ 0 & 0 & 0 & 0 & 0 & 0 & 0 & 0 & 0 & 0\\ 0 & 0 & 0 & 0 & 0 & 0 & 0 & 0 & 0 & 0\\ 0 & 0 & 0 & b{\beta }_{M2H} & 0 & 0 & 0 & 0 & 0 & 0\\ 0 & 0 & 0 & 0 & 0 & 0 & 0 & 0 & 0 & 0\\ 0 & 0 & 0 & 0 & 0 & 0 & 0 & 0 & 0 & 0\end{array})$$

and

$$V=(\begin{array}{cccccccccc}{\gamma }_{M1}+{\mu }_{M}+\delta  & 0 & 0 & 0 & 0 & 0 & 0 & 0 & 0 & 0\\ -{\gamma }_{M1} & {\mu }_{M}+\delta  & 0 & 0 & 0 & 0 & 0 & 0 & 0 & 0\\ -\delta  & 0 & {\gamma }_{M2}+{\mu }_{M} & 0 & 0 & 0 & 0 & 0 & 0 & 0\\ 0 & -\delta  & -{\gamma }_{M2} & {\mu }_{M} & 0 & 0 & 0 & 0 & 0 & 0\\ 0 & 0 & 0 & 0 & {\gamma }_{H}+{\mu }_{H} & 0 & 0 & 0 & 0 & 0\\ 0 & 0 & 0 & 0 & -(1-\varphi ){\gamma }_{H} & q+{\mu }_{H} & 0 & 0 & 0 & 0\\ 0 & 0 & 0 & 0 & -\varphi {\gamma }_{H} & 0 & q+{\mu }_{H} & 0 & 0 & 0\\ 0 & 0 & 0 & 0 & 0 & 0 & 0 & {\gamma }_{H}+{\mu }_{H} & 0 & 0\\ 0 & 0 & 0 & 0 & 0 & 0 & 0 & -(1-\varphi ){\gamma }_{H} & q+{\mu }_{H} & 0\\ 0 & 0 & 0 & 0 & 0 & 0 & 0 & -\varphi {\gamma }_{H} & 0 & q+{\mu }_{H}\end{array}).$$

The well-known result in^31^ shows that the maximum real part of all eigenvalues of the matrix $F-V$ is negative if and only if the spectral radius of the next generation matrix $\rho (F{V}^{-1}) < 1.$ It is straightforward to obtain that

$$F{V}^{-1}=(\begin{array}{cccccccccc}0 & 0 & 0 & 0 & A{\beta }_{H1M} & B & B & 0 & 0 & 0\\ 0 & 0 & 0 & 0 & 0 & 0 & 0 & 0 & 0 & 0\\ 0 & 0 & 0 & 0 & 0 & 0 & 0 & A{\beta }_{H2M} & C & C\\ 0 & 0 & 0 & 0 & 0 & 0 & 0 & 0 & 0 & 0\\ \displaystyle \frac{b{\beta }_{M1H}{\gamma }_{M1}}{(\delta +{\mu }_{M})(\delta +{\gamma }_{M1}+{\mu }_{M})} & \displaystyle \frac{b{\beta }_{M1H}}{\delta +{\mu }_{M}} & 0 & 0 & 0 & 0 & 0 & 0 & 0 & 0\\ 0 & 0 & 0 & 0 & 0 & 0 & 0 & 0 & 0 & 0\\ 0 & 0 & 0 & 0 & 0 & 0 & 0 & 0 & 0 & 0\\ D & \displaystyle \frac{b{\beta }_{M2H}\delta }{{\mu }_{M}(\delta +{\mu }_{M})} & \displaystyle \frac{b{\beta }_{M2H}{\gamma }_{M2}}{{\mu }_{M}({\gamma }_{M2}+{\mu }_{M})} & \displaystyle \frac{b{\beta }_{M2H}}{{\mu }_{M}} & 0 & 0 & 0 & 0 & 0 & 0\\ 0 & 0 & 0 & 0 & 0 & 0 & 0 & 0 & 0 & 0\\ 0 & 0 & 0 & 0 & 0 & 0 & 0 & 0 & 0 & 0\end{array}),$$

where $A=\frac{b{\gamma }_{H}{\lambda }_{M}{\mu }_{H}}{{\lambda }_{H}{\mu }_{M}({\gamma }_{H}+{\mu }_{H})(q+{\mu }_{H})}$, $D=\frac{b{\beta }_{M2H}({\delta }^{2}{\gamma }_{M2}+\delta {\gamma }_{M1}{\gamma }_{M2}+\delta {\gamma }_{M1}{\mu }_{M}+\delta {\gamma }_{M2}{\mu }_{M})}{{\mu }_{M}(\delta +{\mu }_{M})({\gamma }_{M2}+{\mu }_{M})(\delta +{\gamma }_{M1}+{\mu }_{M})}$, $B=\frac{b{\beta }_{H1M}{\lambda }_{M}{\mu }_{H}}{{\lambda }_{H}{\mu }_{M}(q+{\mu }_{H})}$, $C=\frac{b{\beta }_{H2M}{\lambda }_{M}{\mu }_{H}}{{\lambda }_{H}{\mu }_{M}(q+{\mu }_{H})}$. The eigenvalues of the matrix $F{V}^{-1}$ are given by the roots of the characteristic equation:

$${\lambda }^{6}({\lambda }^{2}-\frac{{b}^{2}{\beta }_{H2M}{\beta }_{M2H}{\gamma }_{M2}{\gamma }_{H}{\lambda }_{M}{\mu }_{H}}{{\lambda }_{H}{\mu }_{M}^{2}(q+{\mu }_{H})({\gamma }_{H}+{\mu }_{H})({\gamma }_{M2}+{\mu }_{M})})({\lambda }^{2}-\frac{{b}^{2}{\beta }_{H1M}{\beta }_{M1H}{\gamma }_{H}{\gamma }_{M1}{\lambda }_{M}{\mu }_{H}}{{\lambda }_{H}{\mu }_{M}(\delta +{\mu }_{M})(q+{\mu }_{H})({\gamma }_{H}+{\mu }_{H})(\delta +{\gamma }_{M1}+{\mu }_{M})})=0.$$

The basic reproduction number ${ {\mathcal R} }_{0}$, defined as the average number of secondary cases arising from an average primary case in an entirely susceptible population, is the spectral radius of $\rho (F{V}^{-1})$^31,32^). Therefore,

$${{\mathscr{R}}}_{01}=\sqrt{\displaystyle \frac{{b}^{2}{\beta }_{H1M}{\beta }_{M1H}{\gamma }_{H}{\gamma }_{M1}{\lambda }_{M}{\mu }_{H}}{{\lambda }_{H}{\mu }_{M}(\delta +{\mu }_{M})(q+{\mu }_{H})({\gamma }_{H}+{\mu }_{H})(\delta +{\gamma }_{M1}+{\mu }_{M})}},\,{{\mathscr{R}}}_{02}=\sqrt{\displaystyle \frac{{b}^{2}{\beta }_{H2M}{\beta }_{M2H}{\gamma }_{M2}{\gamma }_{H}{\lambda }_{M}{\mu }_{H}}{{\lambda }_{H}{\mu }_{M}^{2}(q+{\mu }_{H})({\gamma }_{H}+{\mu }_{H})({\gamma }_{M2}+{\mu }_{M})}}.$$

We have

$${ {\mathcal R} }_{0}=\,{\rm{\max }}\,\{{ {\mathcal R} }_{01},{ {\mathcal R} }_{02}\},$$

and the disease-free equilibrium *E*_0_ is locally stable if ${ {\mathcal R} }_{0} < 1$ and unstable if ${ {\mathcal R} }_{0} > 1.$

${ {\mathcal R} }_{01}$ and ${ {\mathcal R} }_{02}$ are the reproduction numbers of non-mutant strain and mutant strain, respectively. It is obvious that ${ {\mathcal R} }_{01}$ decreases with respect to the mutation rate *δ*, but ${ {\mathcal R} }_{02}$ does not depend on *δ*.

System (1) also has two boundary equilibria ${E}_{1}=({S}_{M1},{E}_{M1},{I}_{M1},0,0,{S}_{H1},{E}_{H1},{I}_{H1}^{N},{I}_{H1},{R}_{H1},0,0,0,0)$ and ${E}_{2}=({S}_{M2},0,0,{E}_{M2},{I}_{M2},{S}_{H2},0,0,0,0,{E}_{H2},{I}_{H2}^{N},{I}_{H2},{R}_{H2})$, which correspond to the ultimate domination of each strain. With the non-zero components of the boundary equilibria ${E}_{j}(j=1,2)$ given below:

$$\begin{array}{ccc}\displaystyle {I}_{M1} & = & \displaystyle \frac{{\gamma }_{M2}}{{\mu }_{M}+\delta }{E}_{M1},\,\displaystyle {I}_{H1}^{N}=\frac{(1-\varphi ){\gamma }_{H}}{q+{\mu }_{H}}{E}_{H1},\,\displaystyle {I}_{H1}=\frac{\varphi {\gamma }_{H}}{q+{\mu }_{H}}{E}_{H1},\\ \displaystyle {S}_{M1} & = & \displaystyle \frac{{\lambda }_{H}{\lambda }_{M}(q+{\mu }_{H})}{b{\beta }_{H1M}{\mu }_{H}{\gamma }_{H}{E}_{H1}+{\lambda }_{H}(q+{\mu }_{H}){\mu }_{M}},\\ \displaystyle {E}_{M1} & = & \displaystyle \frac{{\lambda }_{H}({\mu }_{M}+\delta ){\mu }_{H}({\gamma }_{H}+{\mu }_{H}){E}_{H1}}{b{\beta }_{M1H}{\mu }_{H}{\gamma }_{M1}({\lambda }_{H}-({\gamma }_{H}+{\mu }_{H}){E}_{H1})},\\ \displaystyle {E}_{H1} & = & \displaystyle \frac{{\lambda }_{H}^{2}{\mu }_{M}({\mu }_{M}+\delta )({\gamma }_{M1}+{\mu }_{M}+\delta )({\mu }_{H}+q)[{{\mathscr{R}}}_{01}^{2}-1]}{b{\beta }_{H1M}{\gamma }_{H}{\mu }_{H}[b{\beta }_{M1H}{\lambda }_{M}{\gamma }_{M1}+{\lambda }_{H}({\mu }_{M}+\delta )({\gamma }_{M1}+{\mu }_{M}+\delta )]},\\ \displaystyle {S}_{H1} & = & \displaystyle \frac{{\lambda }_{H}}{{\mu }_{H}}-\frac{{\gamma }_{H}+{\mu }_{H}}{{\mu }_{H}}{E}_{H1},\\  & = & \displaystyle \frac{{\lambda }_{H}^{2}({\gamma }_{M1}+{\mu }_{M}+\delta )({\mu }_{M}+\delta )[b{\beta }_{M1H}{\gamma }_{H}{\mu }_{H}+{\mu }_{M}({\gamma }_{H}+{\mu }_{H})({\mu }_{H}+q)]}{b{\beta }_{H1M}{\gamma }_{H}{\mu }_{H}[b{\beta }_{M1H}{\lambda }_{M}{\gamma }_{M1}+{\lambda }_{H}({\mu }_{M}+\delta )({\gamma }_{M1}+{\mu }_{M}+\delta )]},\end{array}$$

and

$$\begin{array}{ccc}\displaystyle {I}_{M2} & = & \displaystyle \frac{{\gamma }_{M2}}{{\mu }_{M}}{E}_{M2},\,\displaystyle {I}_{H2}^{N}=\displaystyle \frac{(1-\varphi ){\gamma }_{H}}{q+{\mu }_{H}}{E}_{H2},\,\displaystyle {I}_{H2}=\displaystyle \frac{\varphi {\gamma }_{H}}{q+{\mu }_{H}}{E}_{H2},\\ \displaystyle {S}_{M2} & = & \displaystyle \frac{{\lambda }_{H}{\lambda }_{M}(q+{\mu }_{H})}{b{\beta }_{H2M}{\mu }_{H}{\gamma }_{H}{E}_{H2}+{\lambda }_{H}(q+{\mu }_{H}){\mu }_{M}},\\ \displaystyle {E}_{M2} & = & \displaystyle \frac{{\lambda }_{H}{\mu }_{M}{\mu }_{H}({\gamma }_{H}+{\mu }_{H}){E}_{H2}}{b{\beta }_{M2H}{\mu }_{H}{\gamma }_{M2}({\lambda }_{H}-({\gamma }_{H}+{\mu }_{H}){E}_{H2})},\\ \displaystyle {E}_{H2} & = & \displaystyle \frac{{\lambda }_{H}^{2}{\mu }_{M}^{2}({\gamma }_{H}+{\mu }_{H})({\gamma }_{M2}+{\mu }_{M})({\mu }_{H}+q)[{{\mathscr{R}}}_{02}^{2}-1]}{b{\beta }_{H2M}{\gamma }_{H}{\mu }_{H}({\gamma }_{H}+{\mu }_{H})[b{\beta }_{M2H}{\lambda }_{M}{\gamma }_{M2}+{\lambda }_{H}{\mu }_{M}({\gamma }_{M2}+{\mu }_{M})]},\\ \displaystyle {S}_{H2} & = & \displaystyle \frac{{\lambda }_{H}}{{\mu }_{H}}-\frac{{\gamma }_{H}+{\mu }_{H}}{{\mu }_{H}}{E}_{H2},\\  & = & \displaystyle \frac{{\lambda }_{H}^{2}({\gamma }_{M2}+{\mu }_{M}){\mu }_{M}[b{\beta }_{M2H}{\gamma }_{H}{\mu }_{H}+{\mu }_{M}({\gamma }_{H}+{\mu }_{H})({\mu }_{H}+q)]}{b{\beta }_{H2M}{\gamma }_{H}{\mu }_{H}^{2}[b{\beta }_{M2H}{\lambda }_{M}{\gamma }_{M2}+{\lambda }_{H}{\mu }_{M}({\gamma }_{M2}+{\mu }_{M})]}.\end{array}$$

These explicit formula for the non-trivial components of boundary equilibria led to the following theorem.

### **Theorem 2**.**1**

*If* ${ {\mathcal R} }_{01} > 1$, *the non-mutant strain prevalent equilibrium* ${E}_{1}$ exists. *If* ${ {\mathcal R} }_{02} > 1,$ *then the mutant strain prevalent equilibrium* ${E}_{2}$ *exists*.

**Figure 2:**
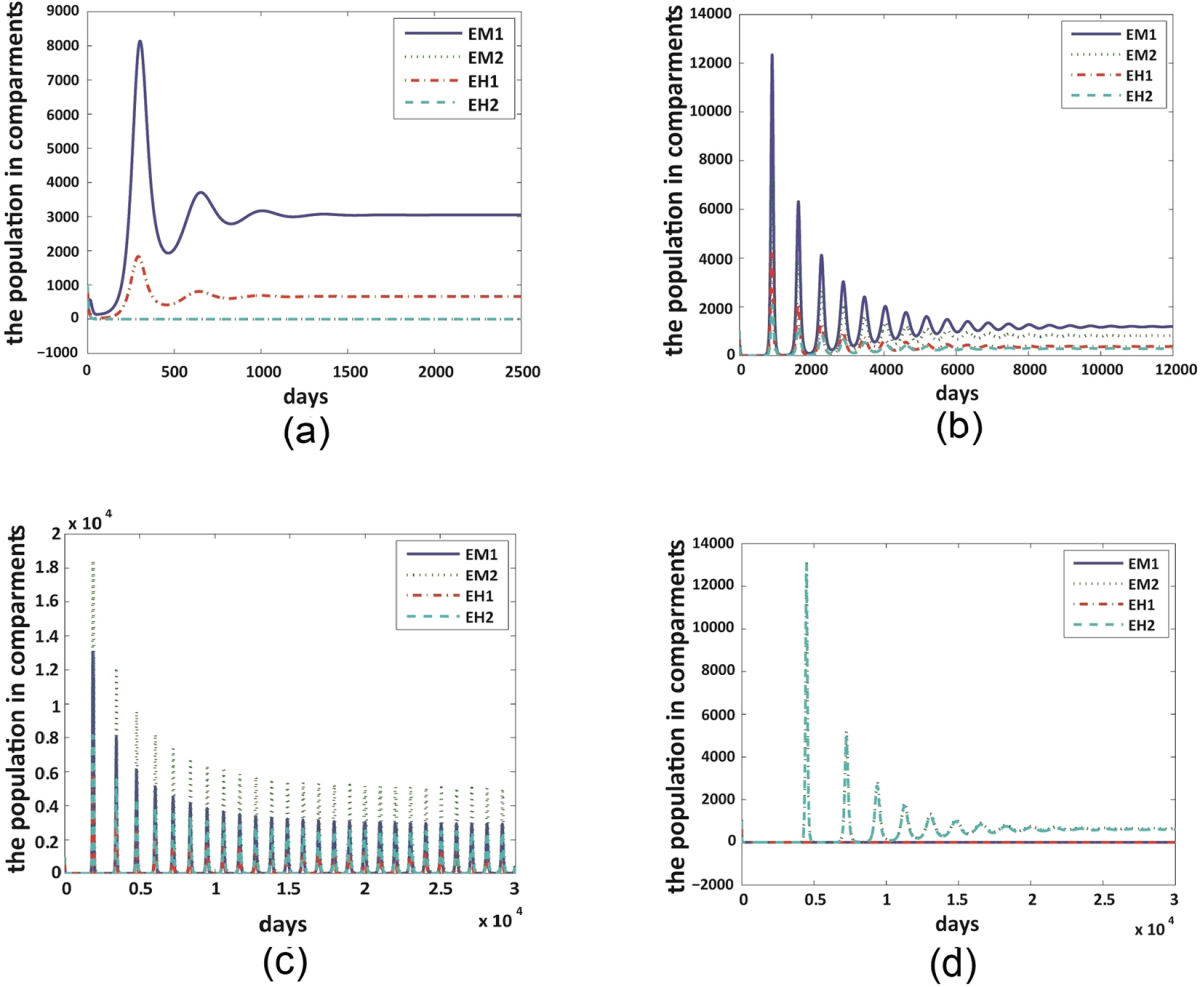
Variations of dominant strain as *δ* increases. We set ${\beta }_{H1M}=0.42,{\beta }_{M1H}=0.3926,{\beta }_{H2M}=0.977,$ ${\beta }_{M2H}=0.726$ for all figures. (**a**) $\delta =0$ and ${ {\mathcal R} }_{01}=15.4216,{ {\mathcal R} }_{02}=3.1985$; (**b**) $\delta =0.1$ and ${ {\mathcal R} }_{01}=7.3270,$ ${ {\mathcal R} }_{02}=3.1958$; (**c**) $\delta =0.2$ and ${ {\mathcal R} }_{01}=4.9616,{ {\mathcal R} }_{02}=3.1985$; (**d**) $\delta =0.6$ and ${ {\mathcal R} }_{01}=2.2123,{ {\mathcal R} }_{02}=3.1985$. Detailed figure legends: ${E}_{M1}$ - the exposed mosquitoes with non-mutant strains; ${E}_{M2}$ - the exposed mosquitoes with mutant strains; ${I}_{M1}$ - the infected mosquitoes with non-mutant strains; ${I}_{M2}$ - the infected mosquitoes with mutant strains.

**Figure 3:**
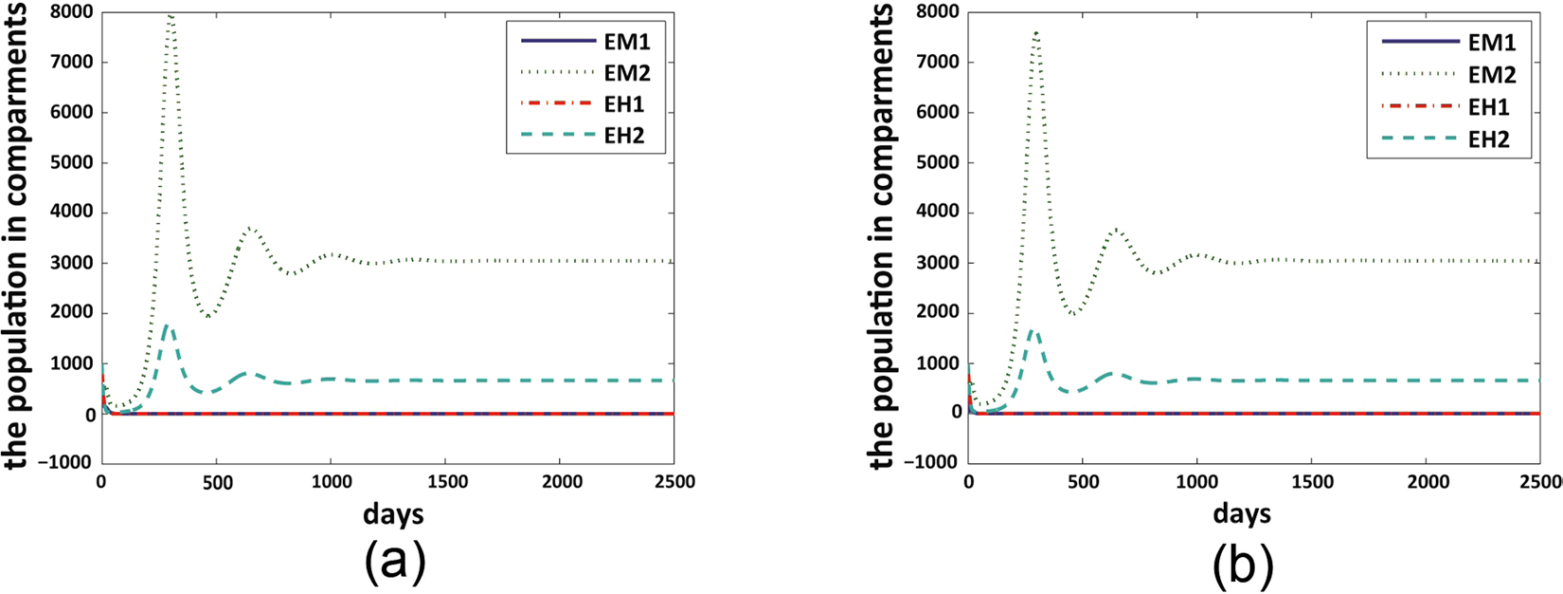
Mutant strain dominates for all *δ* values. We set ${\beta }_{H1M}=0.977,{\beta }_{M1H}=0.726,{\beta }_{H2M}=0.42,$ ${\beta }_{M2H}=0.3926$ for both figures. (**a**) $\delta =0$ and ${ {\mathcal R} }_{01}=3.1985,{ {\mathcal R} }_{02}=15.4216$; (**b**) $\delta =0.3$ and ${ {\mathcal R} }_{01}=0.7829,$ ${ {\mathcal R} }_{02}=15.4216.$ Figure legends are the same with those illustrated in Fig. 2.

**Figure 4:**
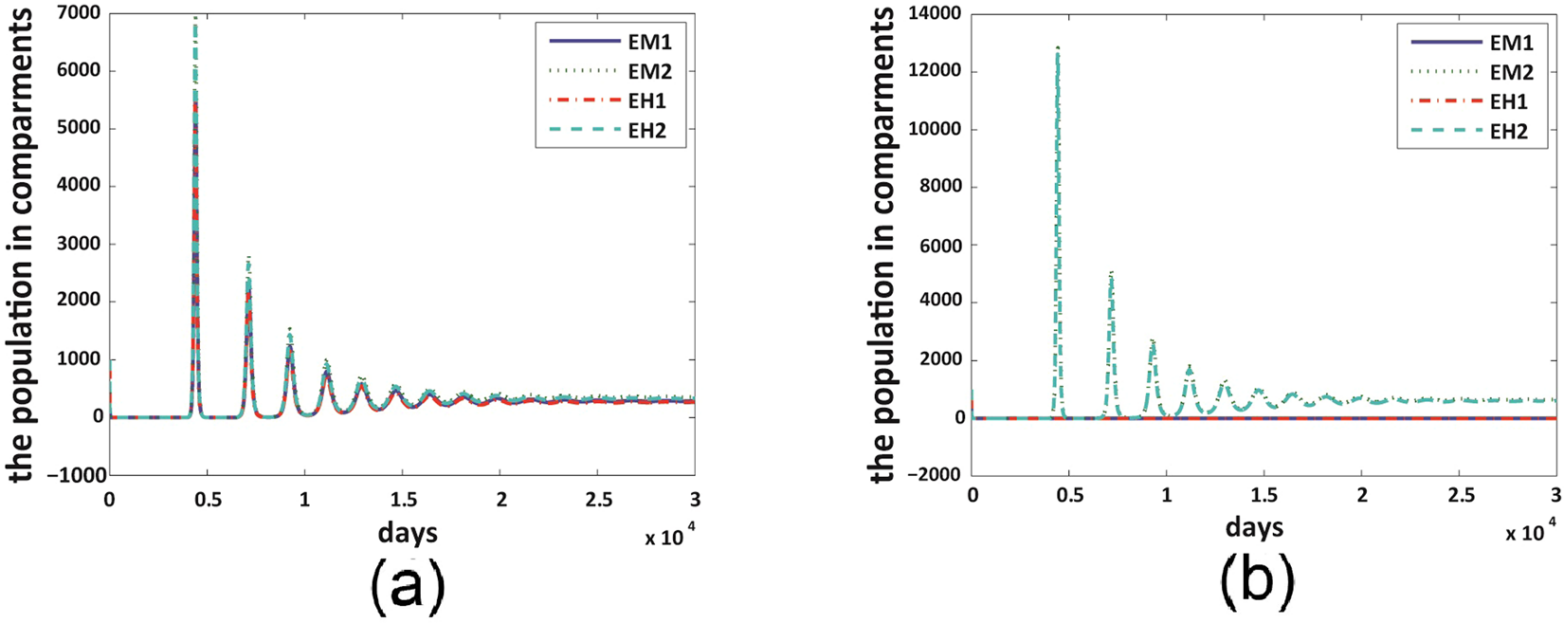
Co-existence happens with no mutation dynamics, but mutant strain dominates when *δ* perturbs from 0. We set ${\beta }_{H1M}={\beta }_{H2M}=0.977,{\beta }_{M1H}={\beta }_{M2H}=0.726$ for both figures. (**a**) $\delta =0$ and ${ {\mathcal R} }_{01}={ {\mathcal R} }_{02}\,=$ $3.1985$. (**b**) $\delta =0.02$ and ${ {\mathcal R} }_{01}=2.5758,{ {\mathcal R} }_{02}=3.1985.$ Figure legends are the same with those illustrated in Fig. 2.

The explicit formula for the positive equilibria with co-existence of both strains for system (1) is hard to derive algebraically. We thus seek to gain our insights from numerical simulations for the existence and stability of the positive equilibria, the boundary equilibria and possible periodic oscillations. We will mainly discuss three cases about the mutation dynamics $(i)$ the infectivity from mosquitoes to humans of the mutant strain is smaller comparing with that of the non-mutant strain, *i*.*e*., ${\beta }_{M1H} < {\beta }_{M2H}$ (shown in Fig. 2); $(ii)$ the infectivity of the mutant strain is larger than that of the non-mutant strain, *i*.*e*., ${\beta }_{M1H} > {\beta }_{M2H}$ (shown in Fig. 3); $(iii)$ the infectivity of the mutant strain is the same as that of the non-mutant strain, *i*.*e*., ${\beta }_{M1H}={\beta }_{M2H}$ (shown in Fig. 4).

For the following numerical simulations, we fix $b=5$, ${\lambda }_{M}=50000$, ${\mu }_{H}=0.00005479$, ${\mu }_{M}=0.0476$, ${\lambda }_{H}=100$, $\varphi =0.85$, $q=0.1667$, ${\gamma }_{H}=0.15$, ${\gamma }_{M1}=0.1857$, ${\gamma }_{M2}=0.1857$ and vary the values of *δ*, ${\beta }_{H1M}$, ${\beta }_{M1H}$, ${\beta }_{H2M}$, and ${\beta }_{M2H}$.

Under the scenario in Fig. 2, the non-mutant strain dominates for small mutation rates, co-existence of both strains could occur under moderate mutation rates, and mutant strain dominates for large mutation rates. Figure 3 shows that, if the mutant strain already possesses a higher transmission probability from mosquito to human, then it will dominate at any mutation rates. In the scenario of Fig. 4 where the two strains obtain same level of transmission probability, co-existence happens when there is no mutation, and mutant strain dominates as soon as the mutation rate perturbs from 0. These results indicate that the mutation dynamics in the bodies of *Aedes albopictus* mosquitoes could result in a progressive take-over of the non-mutant strain by the mutant strain in the human population level in the long run.

## Fitting the Italy 2007 outbreak data

*Aedes albopictus* was introduced in 1990 to Italy and has since been established in urban settings, and was identified as the principal vector for the 2007 CHIKV outbreak in Italy. The mutant strain ECSA-V is believed to be associated to this outbreak^33^, moreover, the co-existence of mutant and nonmutant CHIKV has been reported in this outbreak^34^. We thus use our model to fit this outbreak data and investigate the role played by the mutation dynamics.

**Figure 5:**
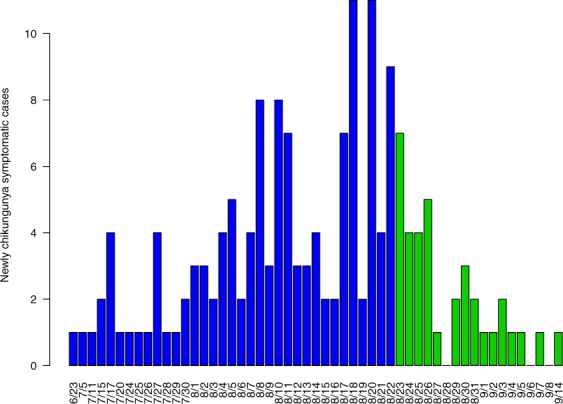
Reported chikungunya cases from June 23rd to September 14th, 2007 in Castiglione di Cervia and Castiglione di Ravenna. Control measures were implemented on August 23rd, so we color the cases reported before interventions blue, and color the cases reported after interventions green. The data depicted above come from G. Rezza *et al*. *Lancet 2007; 370: 1840*–*1846*^44^.

Figure 5 shows the reported case numbers between June 23rd and September 14th, 2007, where the blue bars up to day August 22nd represent the initial outbreak phase before the start of public health implementations. Since the control measures will greatly alter the model parameters, we fit the outbreak data from June 23rd to August 22nd.

Due the short epidemic time scale in comparison to the demographic time scale, we do not consider the natural growth of human population and set ${\lambda }_{H}=0,{\mu }_{H}=0$ in model (1). Further, the outbreak happened mainly in two small villages Castiglione di Cervia and Castiglione di Ravenna (Ravenna province, in northeastern Italy). Therefore, we assume the human population as a constant ${N}_{H}$. This simplifies (1) to model (S1) as shown in the Supplementary material.

For the purpose of comparison, we consider a corresponding model with no mutation dynamics, we set $\delta =0$ and our model (S1) reduces to model (S2), which was used to understand the 2006 Réunion Island outbreak in^24^. We would like to see if incorporating the mutation dynamics and fitting the data would lead to any substantial differences in the estimation of the basic reproductive number.

### Parameters Estimation

All parameter definitions, ranges and relevant references in model (S1) are shown in Table 1. The differences between the mutant and non-mutant strains are reflected in the following parts of the parameterization.

1. Vector competence: the intrinsic ability of a vector to support viral replication so that the virus disseminates from the midgut to the salivary glands for transmission during subsequent vector blood meals. The ECSA-V mutant strain can enhance the replication and the dissemination of CHIKV hence led a significant increase in CHIKV infectivity from *Ae*.*albopictus* to human during transmission^11,12,35,36^. Therefore, we assume the lower bound of ${\beta }_{M2H}$ being larger than that of ${\beta }_{M1H}$ in our sampling (S1);
2. Extrinsic incubation period (EIP): the duration for a mosquito to become infectious since it has taken a viremic blood meal. With relatively short life span of mosquitoes, it is well known that longer EIPs reduce transmission efficiency simply because fewer mosquitoes can live long enough to transmit the virus. However, experimental results indicate that the new ECSA-V variant could shorten the EIP^11,12^. Therefore, we assume $1/{\gamma }_{M2}=2$ days and $1/{\gamma }_{M1}=4$ days. Sensitivity analysis performed in the next subsection shows robustness of the model outcome under a reasonable broad range of the EIP values.

The human population number in the reported area is ${N}_{H}=3968$. The index case is believed to be imported from Kerala, India, which was recorded on June 23rd, 2007 after the arrival in Italy on June 21st. We thus fix the initial values as ${S}_{H}(0)=3968,$ ${E}_{H1}(0)=0,$ ${I}_{H1}^{N}(0)=0,$ ${I}_{H1}(0)=1,$ ${E}_{H2}(0)=0,$ ${I}_{H2}^{N}(0)=0,$ ${I}_{H2}(0)=0.$ We assume that the virus did not circulate in the mosquito population before the outbreak, so the initial values of the mosquito population compartments are set to be zero except for the susceptible mosquito ${S}_{M}\mathrm{(0)}$. It is always hard to know the actual size of mosquito population, so we set ${S}_{M}\mathrm{(0)}$ as one of the model parameters that needs to be fitted.

### Fitting Method

Denote $Y(t)$ as the cumulative symptomatic cases at time $t$, then $\frac{d}{dt}Y(t)=\varphi {\gamma }_{H}{E}_{H1}+\varphi {\gamma }_{H}{E}_{H2}$. We regard the reported symptomatic cases as a random variable follows Poisson-distribution, and fit our model to real data by sampling the posterior distribution of the parameter vector

$$\theta |{\bf{y}}=\{{\beta }_{H2M},{\beta }_{M2H},{\beta }_{H1M},{\beta }_{M1H},{\lambda }_{M},\delta ,{S}_{M}\mathrm{(0)\}|}{\bf{y}}$$

where the vector **y** is derived from $\frac{d}{dt}Y(t)$ by integrating the system of equations for randomly-sampled values of the parameters. To carry out the Markov chain Monte Carlo (MCMC) procedure, we used an adaptive Metropolis-Hastings (M-H) algorithm. The posterior density is

$${f}_{{\rm{\Theta }}|{\bf{y}}}={{\rm{\Pi }}}_{T} {\mathcal L} (Y(t)|\theta ){f}_{{\rm{\Theta }}}\theta .$$

**Figure 6:**
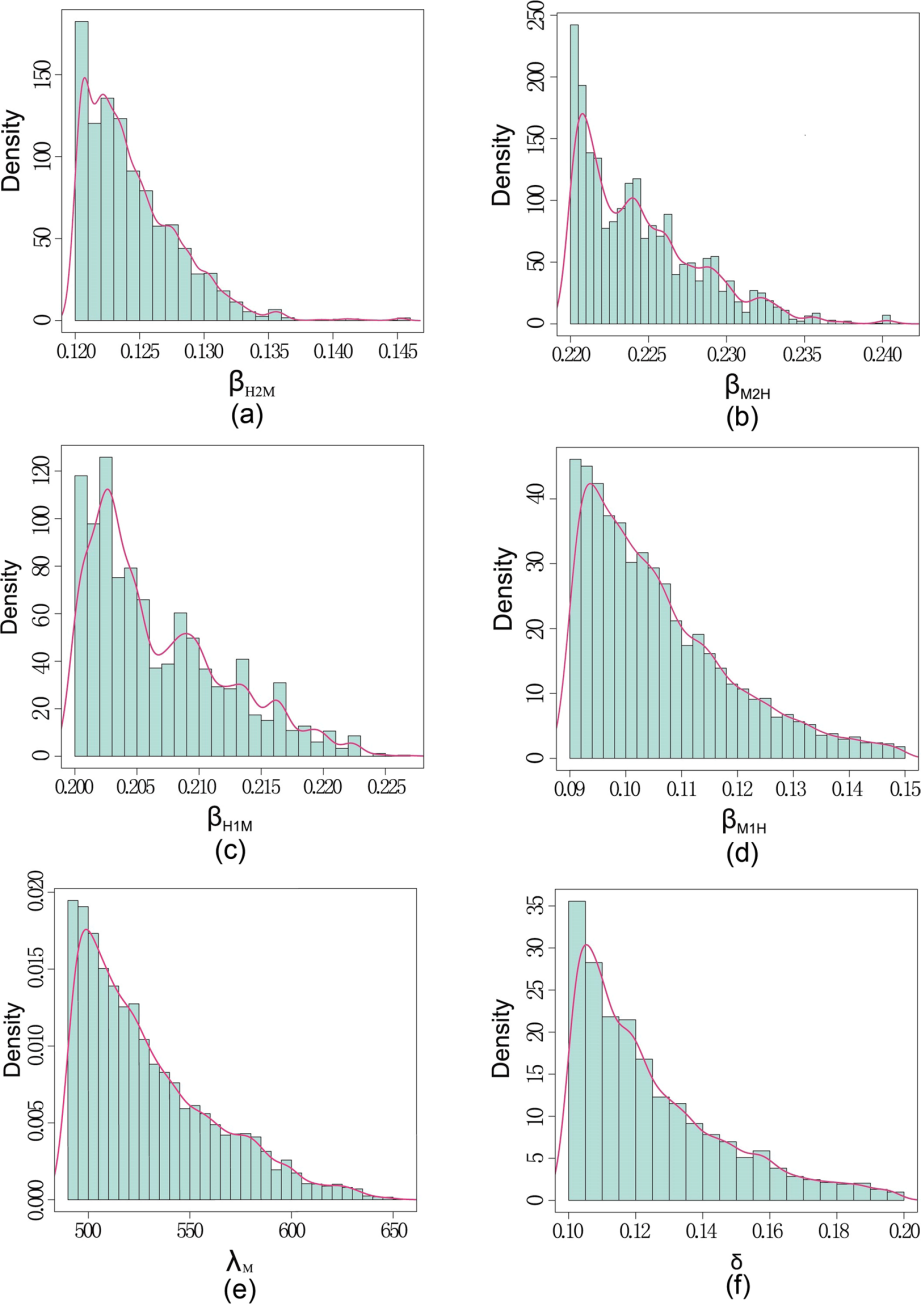
Frequency distribution histograms and probability density curves of the estimated parameters ${\beta }_{H2M},{\beta }_{M2H},{\beta }_{H1M},{\beta }_{M1H},{\lambda }_{M},\delta .$ The blue bars represent frequency distribution histograms and pink lines represent probability density curves.

The algorithm was run 50,000 iterations and we discarded the first 40,000 samples as a burn-in period. The prior density ${f}_{{\rm{\Theta }}}\theta$ is the joint probability of several univariate priors. Of these, ${\beta }_{H2M},{\beta }_{M2H},{\beta }_{H1M},{\beta }_{M1H},{\lambda }_{M},\delta$ are assumed as uniform distributed in the ranges shown in Table 1 and ${S}_{M}\mathrm{(0)}$ being strictly positive. The median and confidence interval of each estimated parameter are listed in Table 2 and their frequency distribution histograms and probability density curves are shown in Fig. 6.

**Table 2:** Parameter values for point estimation and 95% interval estimation in model (S1).

Parameter	Point estimation	95% confidence interval
${\beta }_{H2M}$	0.1237	[0.1201, 0.1320]
${\beta }_{M2H}$	0.2238	[0.2200, 0.2327]
${\beta }_{H1M}$	0.2063	[0.2000, 0.2211]
${\beta }_{M1H}$	0.1025	[0.0900, 0.1285]
${\lambda }_{M}$	522	[490.9350, 597.8843]
$\delta$	0.1215	[0.1007, 0.1784]
${S}_{M0}$	40121	[39712.74, 40401.23]

**Figure 7:**
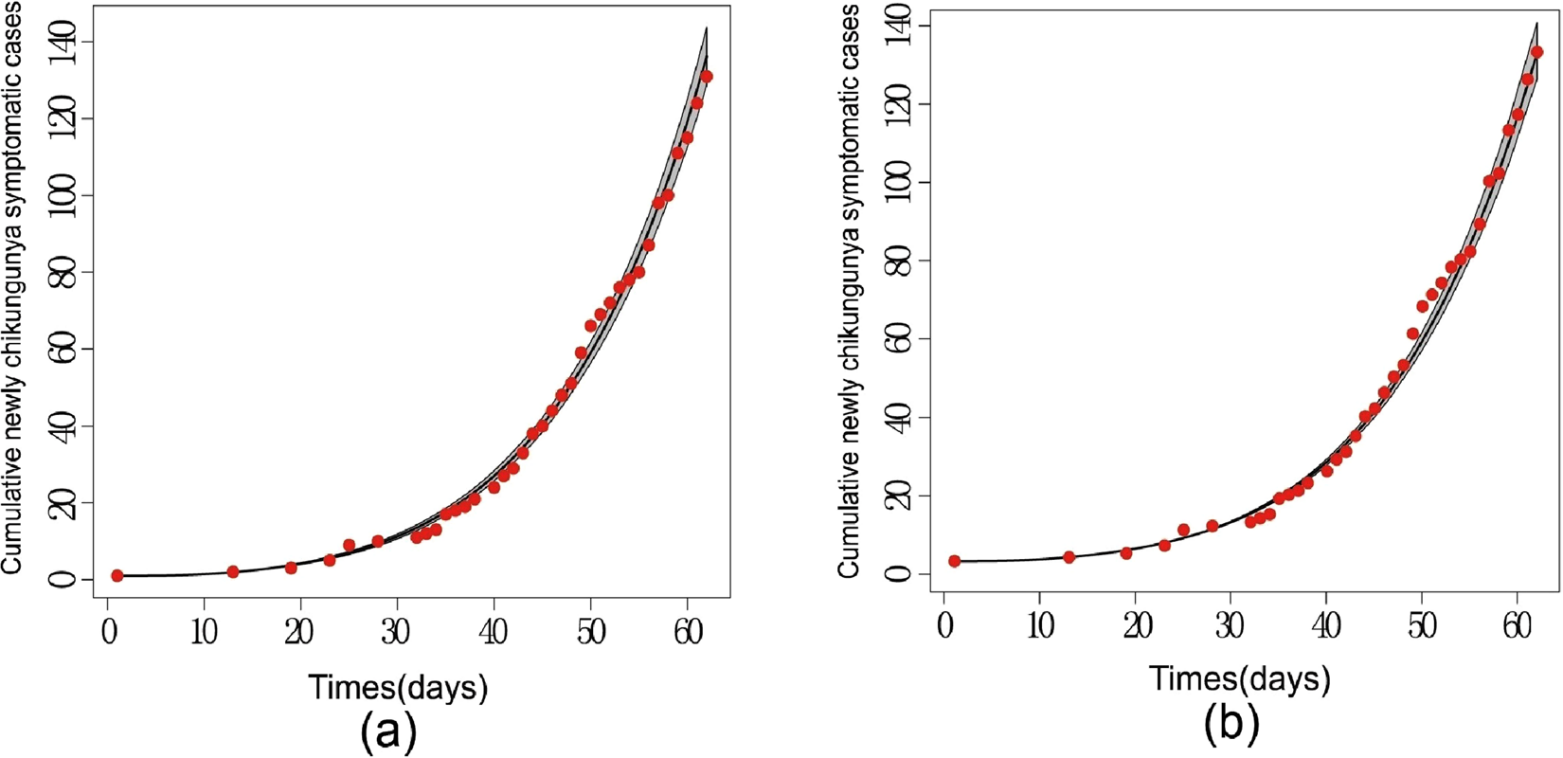
(**a**) The cumulative number of newly chikunkunya symptomatic cases and fitted curve. Red dots represent observed data points while the black solid curve shows the median value based on 5000 simulations by using model (S1), and shaded areas show 95% confidence interval around model (S1) fitted. (**b**) The cumulative number of newly chikunkunya symptomatic cases and fitted curve by using model (S2), as in (**a**).

By using the same method, we fit model (S2) and estimate the four parameters as shown in Table 3. Figure 7 shows the fitting of the same data set with model (S1) and (S2), respectively, in which the shaded band areas represent the 95% confidence interval for fitted parameter values in 5000 runs.

**Table 3:** Parameter values for point estimation and 95% interval estimation in model (S2).

Parameter	Point estimation	95% confidence interval
${\beta }_{H1M}$	0.2387	[0.2361, 0.2399]
${\beta }_{M1H}$	0.1242	[0.1227, 0.1249]
${\lambda }_{M}$	440	[435.2, 449]
${S}_{M0}$	52174	[51986.33, 52359.72]

To see if model (S1) with mutation dynamics gives a better fitting than model (S2), we compare the Akaike information criterion (AIC) to judge the relative quality between the two models:

$${\rm{AIC}}=n\,\mathrm{ln}({\rm{RSS}})+2k,$$

where *n* denotes the sample size and *k* denotes the number of parameters. ${\rm{RSS}}={\sum }_{i=1}^{n}\,{({y}_{i}-f({x}_{i},\hat{\theta }))}^{2}$ is the residual sum of squares. In addition, we also measure the mean absolute percentage error (MAPE) and the root mean square percentage error (RMSPE), which are critical evaluation indicators used to assess the fitting effect and the precision of models. The MAPE and the RMSPE are defined as

$${\rm{MAPE}}=(\frac{1}{n}\sum _{q=2}^{n}|\frac{W{(q)}^{\ast }-W(q)}{W{(q)}^{\ast }}|)\times 100 \% ,$$

$${\rm{RMSPE}}=\sqrt{\frac{{\sum }_{q\mathrm{=2}}^{n}{[(W{(q)}^{\ast }-W(q))/W{(q)}^{\ast }]}^{2}}{n-1}}\times \mathrm{100 \% ,}$$

where $W{(q)}^{\ast }$ is the real value at time $q,$ $W(q)$ is its fitting value and $n$ is the number of data used for prediction. AIC, MAPE and RMSPE are shown in Table 4, and we can conclude that our model with mutation dynamics fits better than the simple model as (S1) has a much lower AIC value.

**Table 4:** The index of the goodness in model fitted.

Model	AIC	MAPE	RMSPE
S1	221.2272	7.715%	10.7696%
S2	229.9071	7.929%	10.7719%

### Calculation of Basic Reproduction Number

**Figure 8:**
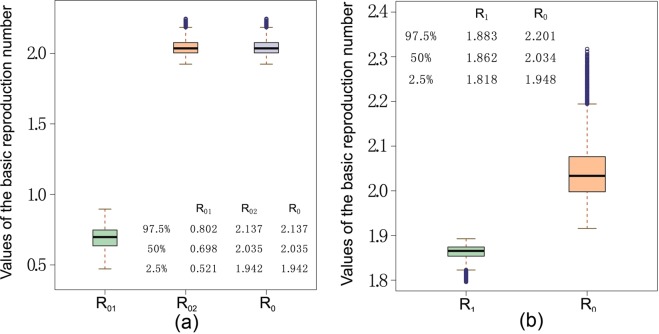
Box plots for the basic reproduction numbers obtained from MCMC sampling. The top of the upper whisker, top of the box, bottom of the box, and bottom of the lower whisker respectively represent the maximum, third quartile, first quartile, and the minimum values of the reproduction numbers calculated from all sampled parameter combinations. (**a**) The box plot of the basic reproduction numbers ${ {\mathcal R} }_{0}$ for model (S1) and that of the non-mutant strain (${ {\mathcal R} }_{01}$) and mutant strain (${ {\mathcal R} }_{02}$). (**b**) The box plot of the basic reproduction numbers ${ {\mathcal R} }_{1}$ for model (S2) and ${ {\mathcal R} }_{0}$ for model (S1).

Based on the estimated parameter values from the MCMC method, we calculate the corresponding uncertainty on the basic reproduction numbers from the sampled parameters. The median and confidence interval of the distribution of the basic reproduction numbers (see Fig. 8(a)) are 2.035 (95% CI: [1.9424, 2.1366]) for ${ {\mathcal R} }_{0},$ 0.698 (95% CI: [0.5213,0.8017]) for ${ {\mathcal R} }_{01},$ 2.035 (95% CI: [1.9424, 2.1366]) for ${ {\mathcal R} }_{02}$. This means that based on our assumption of incorporating the linear mutation rate among mosquitoes, our data fitting results show that the CHIKV outbreak in Italy is majorly associated to the mutant strain, and there should be no outbreak of the non-mutant strain.

Moreover, we obtained that the median and confidence interval of the distribution of the basic reproduction numbers ${ {\mathcal R} }_{1}$ for model (S2) as 1.862 (95% CI: [1.8181, 1.8858]). Figure 8(b) compares the basic reproduction numbers between the two models, ${ {\mathcal R} }_{1}$ and ${ {\mathcal R} }_{0}$, and we observe a significant underestimation if the mutant dynamics is not considered.

### Sensitivity Analysis and Intervention Priorities

**Figure 9:**
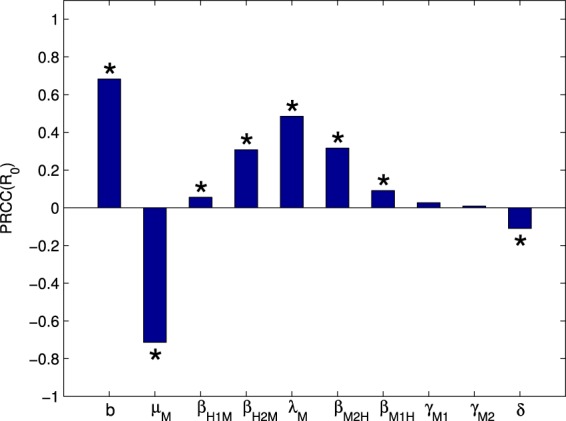
The partial rank correlation coefficient (PRCC) of the basic reproduction number ${ {\mathcal R} }_{0}$ in model (S1) with respect to some model parameters. For each parameter, the absolute value of its PRCC represents the sensitivity of the parameter - the larger the value is, the more sensitive ${ {\mathcal R} }_{0}$ is to the corresponding parameter. * denotes the value of PRCC which is not zero significantly, where the significance level is 0.05.

We perform sensitivity analysis for model (S1) by using the Latin Hypercube Sampling method^37^ to generate 5,000 parameter combinations with each parameter uniformly distributed in its range in Table 1. Figure 9 shows the PRCC values^38^ of these parameters, and we conclude that the basic reproduction number ${ {\mathcal R} }_{0}$ is most sensitive to the mosquito biting and mortality rates; and is more sensitive to the mosquito transmission rate than the EIP; and is more sensitive to the transmission rate of the mutant strain than that of the non-mutant strain.

**Figure 10:**
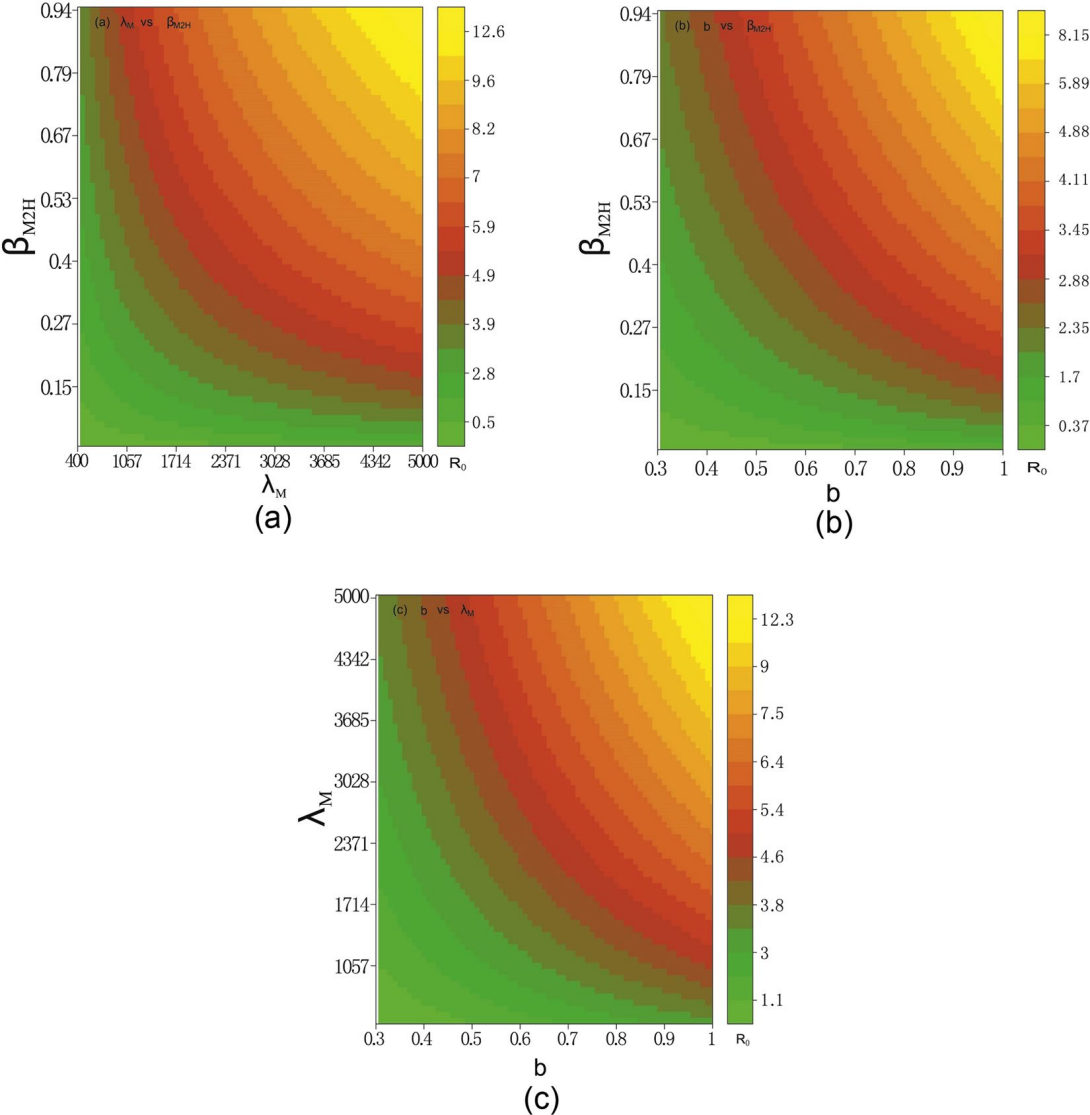
The contour plot of the basic reproduction number in terms of some controllable parameters and other parameter values are given in Table 1. (**a**) ${\beta }_{M2H}$ (transmission rate of mutant strain from mosquito to human) and ${\lambda }_{M}$ (mosquito recruitment rate) (**b**) ${\beta }_{M2H}$ and b (mosquito biting rate) (**c**) ${\lambda }_{M}$ and b. Figure (**a**) and (**b**) show that simultaneously reducing the mosquito recruitment rate and the transmission rate of the mutant strain, and simultaneously reducing the mosquito biting rate and the transmission rate of the mutant strain, can both help with controlling the outbreak. On the other hand, as in Figure (**c**), intervention strategies that only contain the reduction of mosquito recruitment rate and the mosquito biting rate are not efficient in terms of eliminating the outbreak.

To illustrate the dependence of the basic reproduction number ${ {\mathcal R} }_{0}$ in model (S1) on the sensitive and controllable model parameters, we set ${\mu }_{M}=1/28$, ${\beta }_{M1H}=0.1025$, ${\beta }_{H1M}=0.2063$, ${\beta }_{H2M}=0.1237$, $\delta =0.1215$, ${\gamma }_{M1}=1/4$, ${\gamma }_{M2}=1/2$, ${\gamma }_{H}=1/3,\varphi =0.85,q=1/6$ and vary the three controllable parameters ${\beta }_{M2H}=0.2238$ or in $(0.02,0.94),$ $b=0.5$ or in $(0.3,1)$ and ${\lambda }_{M}=522$ or in $(400,5000).$ We produce three contour plots of ${ {\mathcal R} }_{0}$ in terms of ${\beta }_{M2H}$ and ${\lambda }_{M},$ ${\beta }_{M2H}$ and $b,$ $b$ and ${\lambda }_{M}$ in Fig. 10).

Figure 10(a,b) show that reducing either the ${\lambda }_{M}$ and ${\beta }_{M2H}$ pair, or the $b$ and ${\beta }_{M2H}$ pair, will help bring ${ {\mathcal R} }_{0}$ down below 1, hence prevent the outbreak from happening. On the other hand, as shown in Fig. 10(c), intervention strategies that only reducing the mosquito recruitment and biting rates are not efficient for eliminating the outbreak.

## Conclusion and Discussion

The ECSA-V mutant strain was first discovered in the 2005–2006 chikungunya fever outbreak in La Réunion. In particular, the mutation was not detected during the first outbreak wave before September 2005, but was detected during the second wave from December 2005 which involved as many as ten times of the cases reported in the first wave^39^. It is believed that the mutant strain could have been spread by travelers later to Italy, as study confirmed the co-circulation of the non-mutant and mutant strains in the 2007 Chikungunya fever outbreak in Italy^34^. This motivates us in extending a SEIR-type model based on classic mathematical epidemical theory^40^ to examine the impact of virus mutation. We obtained estimates of the basic reproduction number by using some parameters from published literature and our estimation shows that if the mutant strain was involved in the 2007 outbreak, then the disease burden could be underestimated.

The possible high infectivity possessed by the mutant CHIKV strains will not only increase the risk of infection and epidemic size, but could also initiate and sustain chikungunya fever outbreak. In our data fitting, the basic reproduction number caused by non-mutant strain is 0.698 (95%CI: [0.5213,0.8017]), but the estimated value by the mutant strain is 2.035 (95% CI: [1.9424, 2.1366]). Our simulations on varying some controllable model parameters show that the transmission rate of mutant strain from mosquito to human is an important parameter in the control of the disease spread. Some biological studies demonstrated that the adaptation in the *Aedes albopictus* mosquito vector due to the mutation may be associated with evolutionary success, which could further complicate the outbreak control. More seriously, ECSA-V can be seen as the initial step of adaptive mutation of the CHIKV, the second step of adaptive mutation has already been reported^36,41^. Thus new epidemiological evidence, public health interventions, and modeling studies are needed in case the evolution of strain mutations can further facilitate the disease transmission and persistence.

We sampled the virus mutation rate *δ* in a large range $[0,1]$ when fitting our model to the 2007 Italy outbreak data, and obtained a credible range of *δ* as $[0.1007,0.1784]$. This estimation matches what have been observed in an experimental study^42^ - where three out of four *Aedes albopictus* mosquitoes have their saliva samples detected with the mutant strain ten days after infections of the pre-epidemic strain. An estimation of the experimental mutation rate can be calculated easily: assume $M(t)$ as the mosquitoes with their infection dominated by the non-mutant strain and *δ* as the linear mutation rate, then $M^{\prime} (t)=-\,\delta M(t)$ gives the proportion of mosquitoes with non-mutant strain at day 10 as ${e}^{-10\cdot \delta }$ which is nearly 25% from the experiment. Thus we have $\delta \approx 0.1386$ which falls into the estimation of our data fitting result. Indeed, more experiments involving large *Aedes albopictus* populations are needed to enhance the experimental estimation of the mutation potentials.

## Limitations

Our model can be improved if more detailed biological evidence becomes available in the future. Here we would like to discuss some neglected factors in our model and future directions for this study. First of all, although the mutant strain have been discovered in many of the recent chikungunya outbreaks, there is limited knowledge on the mutation mechanism. Our model assumes a linear mutation rate happening among mosquitoes infected with the non-mutant strain, which is only one of the many possibilities. Thus future work should consider and model other possible mutation dynamics, fitting models with various hypothesis to real outbreak data could help select the reasonable ones. Secondly, we use real data to fit parameters with undetermined broad ranges while leaving the other parameters as fixed values including mosquito biting rate and the proportion of symptomatic individuals. However, the biting rate varies in *Aedes* species and could also depend on temperature and urbanization, and the proportion of symptomatic cases is always difficult to estimate on the population level. Lastly, our model structure should be adjusted based on further knowledges, such as the cross-immunity between the mutant and non-mutant strains after recovery, waning immunity^43^, and transmission ability difference between symptomatic and asymptomatic individuals.

## Supplementary information

Supplementary information
