# Supplementary material for: Modelling and Analyzing Virus Mutation Dynamics of Chikungunya Outbreaks
Source: Sci Rep. 2019 Feb 27;9:2860. doi: 10.1038/s41598-019-38792-4 (PMC6393467; doi:10.1038/s41598-019-38792-4)
Supplement: Supplementary file 1 — Supplementary information [file 41598_2019_38792_MOESM1_ESM.pdf]

Supplementary Material

Modelling and Analyzing Virus Mutation Dynamics of Chikungunya Outbreaks

Xiaomei Feng, Xi Huo, Biao Tang, Sanyi Tang, Kai Wang, Jianhong Wu

In order to fit the 2007 chikungunya fever outbreak in Italy, we do not consider the human demographic effects, that is, we assume that $\lambda_H = 0, \mu_H = 0$ in model (2.1). Further, since the outbreak happened mainly in two small villages we assume the human population being a constant value N_H. Now model (2.1) is simplified as follows:

$$
\begin{aligned}
\frac{dS_M}{dt} &= \lambda_M - b\beta_{H1M}\frac{S_M(I_{H1}^N + I_{H1})}{N_H} - b\beta_{H2M}\frac{S_M(I_{H2}^N + I_{H2})}{N_H} - \mu_M S_M,\\
\frac{dE_{M1}}{dt} &= b\beta_{H1M}\frac{S_M(I_{H1}^N + I_{H1})}{N_H} - \gamma_{M1}E_{M1} - \mu_M E_{M1} - \delta E_{M1},\\
\frac{dI_{M1}}{dt} &= \gamma_{M1}E_{M1} - \mu_M I_{M1} - \delta I_{M1},\\
\frac{dE_{M2}}{dt} &= b\beta_{H2M}\frac{S_M(I_{H2}^N + I_{H2})}{N_H} - \gamma_{M2}E_{M2} - \mu_M E_{M2} + \delta E_{M1},\\
\frac{dI_{M2}}{dt} &= \gamma_{M2}E_{M2} - \mu_M I_{M2} + \delta I_{M1},\\
\frac{dS_H}{dt} &= -\frac{b\beta_{M1H}S_H I_{M1}}{N_H} - \frac{b\beta_{M2H}S_H I_{M2}}{N_H},\\
\frac{dE_{H1}}{dt} &= \frac{b\beta_{M1H}S_H I_{M1}}{N_H} - \gamma_H E_{H1},\\
\frac{dI_{H1}^N}{dt} &= (1-\phi)\gamma_H E_{H1} - qI_{H1}^N,\\
\frac{dI_{H1}}{dt} &= \phi\gamma_H E_{H1} - qI_{H1},\\
\frac{dE_{H2}}{dt} &= \frac{b\beta_{M2H}S_H I_{M2}}{N_H} - \gamma_H E_{H2},\\
\frac{dI_{H2}^N}{dt} &= (1-\phi)\gamma_H E_{H2} - qI_{H2}^N,\\
\frac{dI_{H2}}{dt} &= \phi\gamma_H E_{H2} - qI_{H2},\\
\frac{dR_{H2}}{dt} &= q(I_{H2}^N + I_{H2}),
\end{aligned}
\tag{S1}
$$

To further simplify the model by considering only nonmutant CHIKV without the mutation possibilities, we assume that $\delta = 0$ and reduce model (S1) to the following model which was used

to understand the 2006 Réunion Island outbreak:

$$\begin{aligned}
\frac{dS_M}{dt} &= \lambda_M - b\beta_{H1M}\frac{S_M(I_{H1}^N + I_{H1})}{N_H} - \mu_M S_M,\\
\frac{dE_{M1}}{dt} &= b\beta_{H1M}\frac{S_M(I_{H1}^N + I_{H1})}{N_H} - \gamma_{M1}E_{M1} - \mu_M E_{M1},\\
\frac{dI_{M1}}{dt} &= \gamma_{M1}E_{M1} - \mu_M I_{M1},\\
\frac{dS_H}{dt} &= -b\beta_{M1H}\frac{S_H I_{M1}}{N_H},\\
\frac{dE_{H1}}{dt} &= b\beta_{M1H}\frac{S_H I_{M1}}{N_H} - \gamma_H E_{H1},\\
\frac{dI_{H1}^N}{dt} &= (1-\phi)\gamma_H E_{H1} - qI_{H1}^N,\\
\frac{dI_{H1}}{dt} &= \phi\gamma_H E_{H1} - qI_{H1},
\end{aligned} \tag{S2}$$

with the basic reproduction number $\mathcal{R}_1 = \sqrt{\frac{b^2\beta_{H1M}\beta_{M1H}\gamma_H\gamma_{M1}\lambda_M}{N_H\mu_M q\gamma_H(\delta+\mu_M)(\delta+\gamma_{M1}+\mu_M)}}$.
